# Identification of key genes and signaling pathways associated with dementia with Lewy bodies and Parkinson's disease dementia using bioinformatics

**DOI:** 10.3389/fneur.2023.1029370

**Published:** 2023-03-09

**Authors:** Jing Xu, Jia Li, Ya-juan Sun, Wei Quan, Li Liu, Qing-hui Zhang, Yi-dan Qin, Xiao-chen Pei, Hang Su, Jia-jun Chen

**Affiliations:** Department of Neurology, China–Japan Union Hospital of Jilin University, Changchun, Jilin, China

**Keywords:** Lewy body dementias, dementia with Lewy bodies, Parkinson's disease dementia, weighted gene co-expression network analysis, hub gene, biomarker

## Abstract

**Objective:**

Dementia with Lewy bodies (DLB) and Parkinson's disease dementia (PDD) are collectively known as Lewy body dementia (LBD). Considering the heterogeneous nature of LBD and the different constellations of symptoms with which patients can present, the exact molecular mechanism underlying the differences between these two isoforms is still unknown. Therefore, this study aimed to explore the biomarkers and potential mechanisms that distinguish between PDD and DLB.

**Methods:**

The mRNA expression profile dataset of GSE150696 was acquired from the Gene Expression Omnibus (GEO) database. Differentially expressed genes (DEGs) between 12 DLB and 12 PDD were identified from Brodmann area 9 of human postmortem brains using GEO2R. A series of bioinformatics methods were applied to identify the potential signaling pathways involved, and a protein–protein interaction (PPI) network was constructed. Weighted gene co-expression network analysis (WGCNA) was used to further investigate the relationship between gene co-expression and different LBD subtypes. Hub genes that are strongly associated with PDD and DLB were obtained from the intersection of DEGs and selected modules by WGCNA.

**Results:**

A total of 1,864 DEGs between PDD and DLB were filtered by the online analysis tool GEO2R. We found that the most significant GO- and KEGG-enriched terms are involved in the establishment of the vesicle localization and pathways of neurodegeneration-multiple diseases. Glycerolipid metabolism and viral myocarditis were enriched in the PDD group. A B-cell receptor signaling pathway and one carbon pool by folate correlated with DLB in the results obtained from the GSEA. We found several clusters of co-expressed genes which we designated by colors in our WGCNA analysis. Furthermore, we identified seven upregulated genes, namely, SNAP25, GRIN2A, GABRG2, GABRA1, GRIA1, SLC17A6, and SYN1, which are significantly correlated with PDD.

**Conclusion:**

The seven hub genes and the signaling pathways we identified may be involved in the heterogeneous pathogenesis of PDD and DLB.

## 1. Introduction

Lewy body dementia (LBD) is the second most prevalent form of neurodegenerative dementia after Alzheimer's disease (AD) in patients older than 65 years (1). Parkinson's disease dementia (PDD) and dementia with Lewy bodies (DLB), collectively known as LBD, are synucleinopathies morphologically characterized by neuronal loss, inclusions containing Lewy body/α-synuclein and β-amyloid, and tau pathologies, often reported as part of the same spectrum (2, 3). Cognitive decline in the LBD may, in part, be due to a general loss of synapses and related functional failure (4, 5). There is an average of 30–40% loss of synapses in the frontal and the temporal cortex in DLB (6), and in PDD, a reduction of the synaptophysin immunoreactivity of the cortical neuropil was 8.2% (7). Synaptic functional failure happens in the early stages of synucleinopathies due to altered transport of vesicles, synaptic proteins, and mitochondria, which lead to presynaptic terminal loss, dendritic damage, axonal dystrophy, and eventually degeneration of selective neuronal populations within the striatonigral and cortico-limbic systems, among others (6). Clinical distinctions between the two refer to the “so-called 1-year rule” (1, 8, 9), that is, the term DLB is used if dementia occurs before or concurrently with parkinsonism or within 1 year of onset of the motor symptoms; PDD describes dementia starting 1 year or more after Parkinson's disease (PD) becomes well-established (1). This mode of distinction is clearly arbitrary and based on the distinction between the time of onset of cognitive and motor symptoms (3). The mechanisms underlying these differences in clinical manifestations are unclear, and it is necessary to explore the mechanisms by which differences occur to differentiate from the early stages of the disease or even from differences at the genetic or molecular level, and hopefully to provide targeted treatments for these differences. Therefore, it is important to further study the differences in the pathogenesis between the two dementias (10). To this end, we used bioinformatics methods to delve deep into the mechanisms of their heterogeneity.

## 2. Materials and methods

### 2.1. Data source

Gene expression datasets were obtained from the GEO database. After a careful review of the datasets, we chose the series of mRNA expression profile datasets of GSE150696 (https://www.ncbi.nlm.nih.gov/geo/query/acc.cgi?acc=GSE150696) (11). Consensus criteria used for clinical diagnoses of PDD and DLB with neuropathologic confirmation have been previously described in detail (12). The samples processed in each group were matched for age, sex, and postmortem interval. The Brodmann area 9 from human postmortem brains was chosen for analysis. A total of 12 (6 women/6 men) PDD samples and 12 (6 women/6 men) DLB samples were retrieved from GSE150696 and published on 24 May 2021. All brain samples were provided by the Brains for Dementia Research, UK. Data were freely available online, and our study did not involve any experiments in the lab performed by any of the authors.

### 2.2. Data processing of differentially expressed genes

The GEO2R online analysis tool (https://www.ncbi.nlm.nih.gov/geo/geo2r/) was used to detect differentially expressed genes (DEGs) between PDD and DLB samples, and the *P-*value and |log (FC)| (FC-fold change) were calculated. Genes that met the cutoff criteria, *P* < 0.05 and |log FC| ≥ 1.0, were considered DEGs (11). Genes with *P* < 0.05 and log FC ≥ 1.0 were considered upregulated genes, and genes with *P* < 0.05 and log FC ≤ −1.0 were considered downregulated genes (11). GraphPad Prism 9 (GraphPad Software, San Diego, CA, USA; www.graphpad.com), graphing software that can perform data analysis and data visualization, was used to visualize volcano maps of all identified DEGs and a heat map of the top 50 genes (11).

### 2.3. GO and KEGG pathway analysis

The R software (version 4.2.1) was used for the GO annotation, the KEGG pathway enrichment analysis, and the visualization of DEGs (13). An online analysis tool Metascape website (http://metascape.org) was used for GO and KEGG analyses of gene modules selected by weighted gene co-expression network analysis (WGCNA) (14).

### 2.4. Gene set enrichment analysis

Gene Set Enrichment Analysis (GSEA) is a promising and widely used software package (15) that derives gene sets to find the different biological functions of the whole genes between PDD and DLB. The potential contribution of the whole altered genes to LBD was explored using the GSEA software (version 4.2.3). A normalized enrichment score (NES) was calculated, and NES is the enrichment score for the gene set after it has been normalized across analyzed gene sets. The gene set was deemed to be significantly enriched when the *P*-value was < 5% and |NES| was >1 for each analysis (16).

### 2.5. Weighted gene co-expression network analysis

Weighted gene co-expression network analysis (WGCNA) can cluster genes with higher co-expression levels, assemble them into modules, and establish connections between their modules and phenotypes to find the hub genes of the phenotype. We selected a WGCNA package of the R software to filter the top 6,000 median absolute deviation genes to construct a representation matrix and the scale-free network (17). The β-value was selected as long as *R*^2^ was >0.8. The β-value was a soft threshold. The algorithm introduces an approximate scale-free topology to accurately calculate the soft threshold and then replaces the hard threshold of the previous traditional algorithm; Scale-free topologies are more realistic when compared with random networks (18). Based on the selected soft threshold, network modules were constructed by clustering the gene topology matrix using the dynamic shear tree algorithm. The minimum number of genes included in the network module was set to 20. The module color was established by using the degree of dissimilarity automatically by WGCNA software (18). The relationships between modules and LBD haplotypes are shown with a heatmap. We measured the module membership (mM) and gene significance (GS) of individual genes, and the hub genes in the selected modules with |mM| >0.8 and |GS| >0.2 were screened for further analysis (19, 20).

### 2.6. Protein–protein interaction network construction and hub gene identification

We used the online Search Tool for the Retrieval of Interacting Genes database tool (STRING-DB) (http://string-db.org/) to analyze protein–protein interaction (PPI) information (21). PPI pairs were extracted with a combined score of >0.4, and the results were calculated with their automatically cited parameters. Subsequently, the PPI network was visualized using the CytoHubba plug-in in Cytoscape software (version 3.9.1; https://www.cytoscape.org/) was used to calculate the degree of each protein node (11). We considered the top 30 identified genes as the hub genes in our study. Finally, we screened the hub genes by intersecting them in the selected modules, mentioned earlier, and in the DEG-PPI network.

## 3. Results

### 3.1. Identification of DEGs

**Figure 1:**
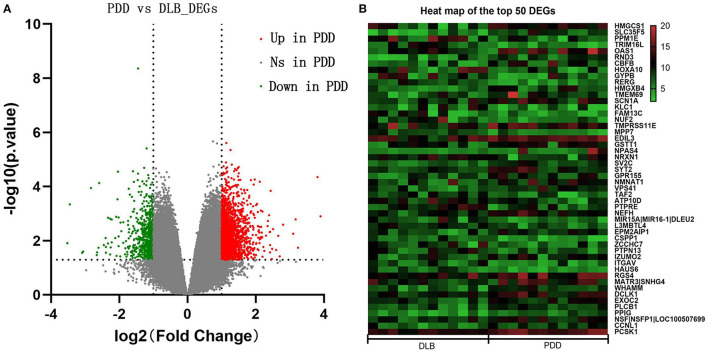
**(A)** Volcano maps of all differentially expressed genes (DEGs) between Parkinson's disease dementia (PDD) and Dementia with Lewy bodies (DLB). The red color represents upregulated genes, and the green color represents downregulated genes in PDD; gray represents genes that are not differentially expressed. The dashed horizontal and vertical axes indicate the log fold change absolute threshold of 1 and the *P*-value threshold of 0.05, respectively. **(B)** Heat map of the top 50 DEGs. The color gradient from green to red indicates gene regulation from downregulation to upregulation in PDD or DLB. Numbers 5–20 indicate gene expression levels.

The samples of PDD and DLB obtained from Brodmann area 9 of postmortem brains were selected for the present study from the GSE150696 series. On the basis of the criteria of *P* < 0.05 and |log FC| ≥ 1.0, 1,864 DEGs between PDD and DLB were filtered by the online analysis tool GEO2R. It included 1240 upregulated and 624 downregulated DEGs in patients with PDD (Supplementary Table S1). A volcano map of all identified DEGs is shown in Figure 1A. In addition, a heat map of the top 50 DEGs is shown in Figure 1B.

### 3.2. Functional and pathway enrichment

#### 3.2.1. Analyses for DEGs

**Figure 2:**
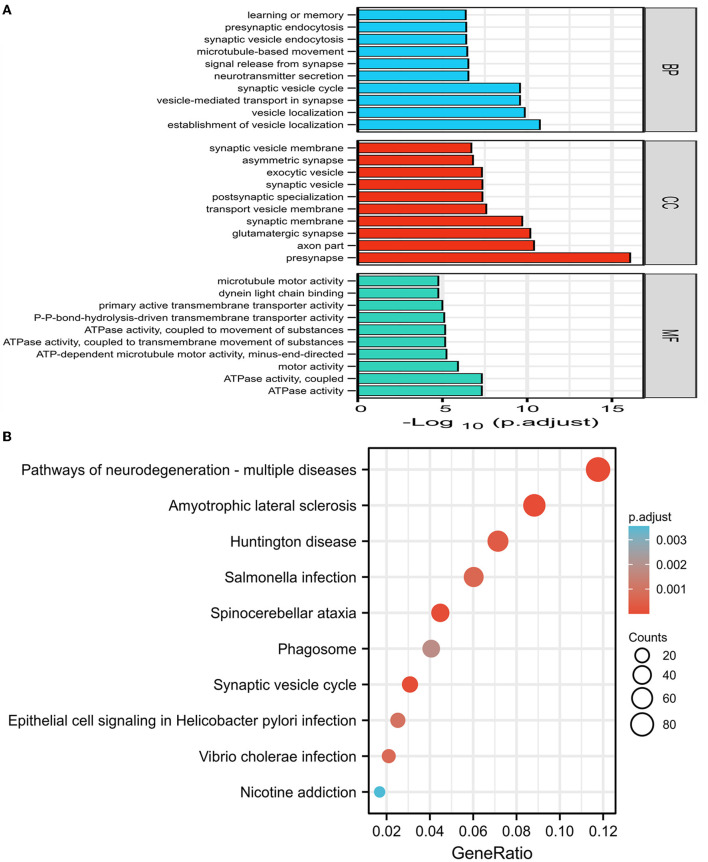
**(A)** The results of gene ontology (GO) enrichment categories of the DEGs for both Parkinson's disease dementia (PDD) and Dementia with Lewy bodies (DLB) groups including biological process (BP), cellular component (CC), and molecular function (MF). **(B)** The results of the Kyoto encyclopedia of genes and genomes (KEGG) pathway enrichment analyses of the DEGs for both PDD and DLB, the abscissa represents GeneRatio (the number of genes enriched in this KEGG entry/the total number of genes), and the ordinate represents KEGG terms. P-adjust refers to the *P*-value after correction, the darker the red color, the greater the correlation. Counts refer to the number of genes enriched in this KEGG entry, the larger the circle, the more the number of genes.

The GO and KEGG enrichment analyses were performed on 1,864 DEGs between PDD and DLB, and the findings were visualized with the cluster profiler package of R software (22). The mainly enriched biological process of GO analysis included the establishment of vesicle localization, vesicle-mediated transport in the synapse, and learning and memory. The cellular component of GO analysis included the presynapse axon part and the glutamatergic synapse. The molecular function of GO analysis included ATPase activity and motor activity (Figure 2A). The results of GO analysis of up and down DEGs between PDD and DLB are shown in Supplementary Figures S1A, S2. In addition, the results of the KEGG pathway analysis showed that DEGs were mainly enriched in pathways in neurodegeneration-multiple diseases, Amyotrophic lateral sclerosis, and Huntington's disease (Figure 2B). The results of the KEGG pathway enrichment analysis in up DEGs are shown in Supplementary Figure S1B (down DEGs were not enriched by the corresponding KEGG pathway).

#### 3.2.2. Gene set enrichment analysis

**Figure 3:**
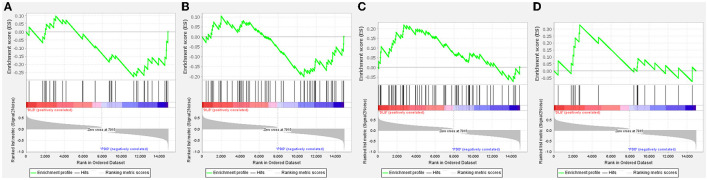
Gene set enrichment analysis (GSEA) plots of the most enriched gene sets in the Parkinson's disease dementia (PDD) and Dementia with Lewy bodies (DLB) groups. The enriched pathways positively correlated with PDD: **(A)** glycerolipid metabolism; **(B)** viral myocarditis. The enriched pathways positively correlated with DLB: **(C)** One carbon pool by folate; **(D)** B-cell receptor signaling pathway.

The GSEA analysis was used to filter unique pathways involved in the pathogenesis of PDD or DLB. When the green line plot was in the negative direction, the gene on the right side of the maximum enrichment score value was the core gene, the pathway was positively correlated with the PDD group, contrarily, the pathway was positively correlated with the DLB group. As shown in Figures 3A–D, the pathways of glycerolipid metabolism and viral myocarditis were positively correlated with the PDD group. The pathways of the B-cell receptor signaling pathway and one carbon pool by folate signaling pathways were positively correlated with the DLB group.

### 3.3. Weighted gene co-expression network analysis

**Figure 4:**
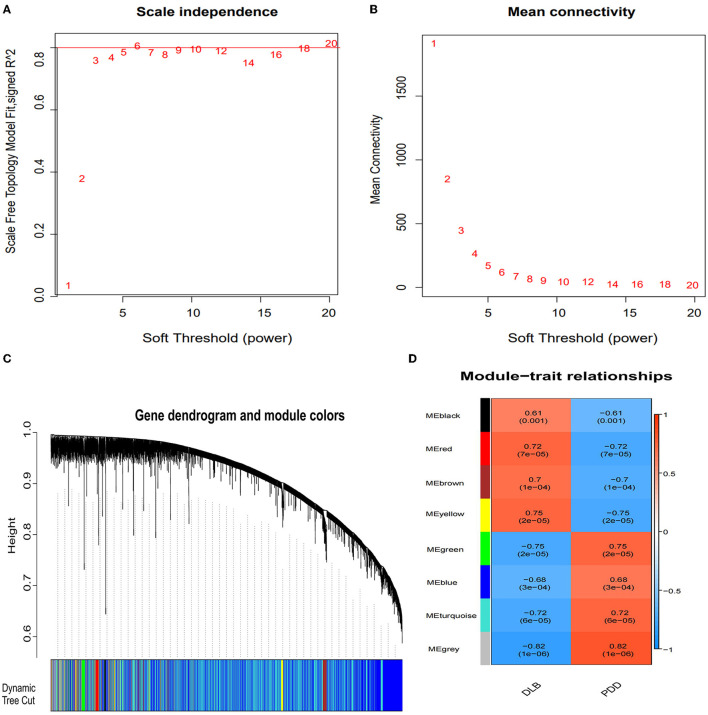
Identification of the gene modules related to Parkinson's disease dementia (PDD) and Dementia with Lewy bodies (DLB) by weighted gene co-expression network analysis (WGCNA). **(A)** Determination of the soft threshold in the WGCNA algorithm. The approximate scale-free fit index can be attained at the soft-thresholding power of 6. **(B)** Mean connectivity of various soft-thresholding powers. **(C)** Clustering dendrograms showing eight modules that contain a group of highly connected genes. Each designated color represents a certain gene module. Diverse colors reflect corresponding modules, and the gray module represents genes that are not assigned to each network. **(D)** The heatmap of the relationship between each gene module and each subtype of LBD. The red color represents a positive correlation, while the blue color represents a negative correlation. Each cell contains the corresponding correlation and *P-*value.

We used WGCNA software to identify the associations between the key gene modules related to PDD and DLB. As shown in Figures 4A, B, the power was set as 6 for further analysis and satisfied the scale-free co-expression network relationships, with the mean value of the adjacency function gradually approaching 0. According to the module-trait relationships, eight modules were identified by the average linkage hierarchical clustering method from the co-expression network, and the colors were defined by the software automatically (Figure 4C). Based on the correlation between different modules and subtypes of LBD shown in the heatmap, we found the green module was significantly positively associated with the PDD group (cor = 0.75, *P*-value < 0.01); the yellow module was significantly positively associated with the DLB group (cor = 0.75, *P*-value < 0.01) (Figure 4D); and the gray module represented genes that were not assigned to each network. Other modules such as black, turquoise, red, brown, and blue also suggest a clear correlation.

### 3.4. Enrichment analyses of module genes identified by WGCNA

**Figure 5:**
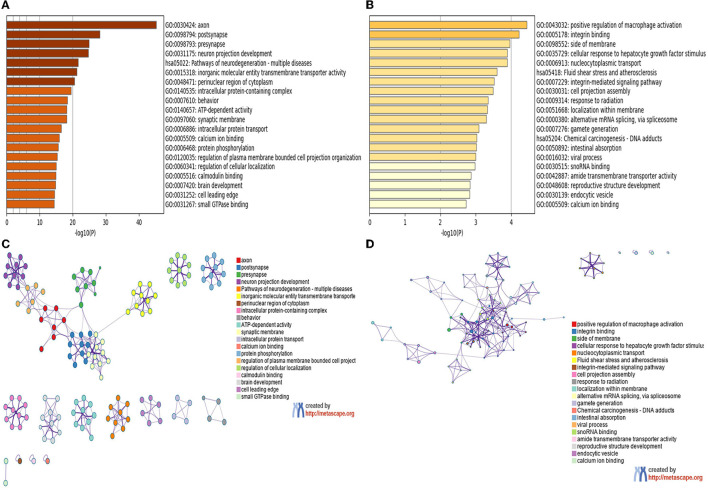
GO and KEGG enrichment analyses of module genes recognized by the WGCNA analysis. **(A)** Blue module. **(B)** Yellow module, bar plots colored by *p*-values. **(C)** Blue module. **(D)** Yellow module, Networks colored by genes.

We used the Metascape tool to perform GO annotation and KEGG pathway enrichment analyses to analyze the features of the module genes. The number of genes within the blue and yellow modules was 2,530 and 182, respectively. As shown in Figures 5A, C, genes in the blue module were mainly involved in the axon, postsynapse, presynapse, neuron projection development, and pathways of multiple neurodegenerative diseases. Genes in the yellow module (Figures 5B, D) were mainly involved in the positive regulation of macrophage activation, integrin binding, side of membrane, and cellular response to hepatocyte growth factor stimulus. The results of GO and KEGG pathway enrichment analyses with regard to the green module are shown in Supplementary Figures S3A, B.

### 3.5. Identification of hub genes

**Figure 6:**
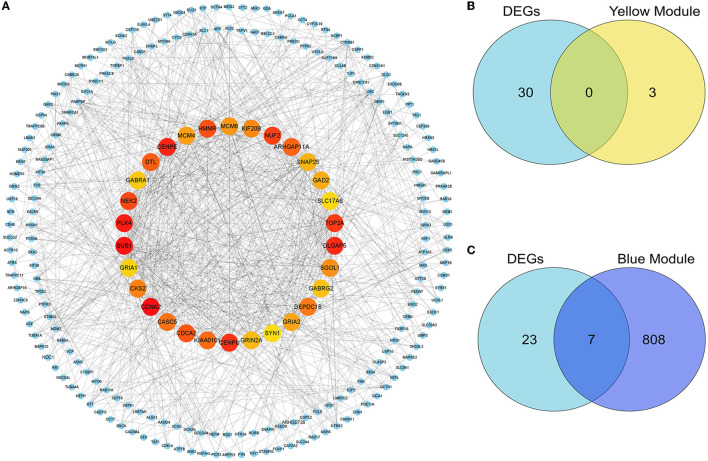
Identification of the hub genes and key genes. The PPI network of the DEGs. **(A)** The top 30 hub genes among DEGs were confirmed with the Cytohubba plugin. The colors of the nodes reflect the degree of connectivity, the darker the yellow color, the more obvious the connectivity is. The key genes are defined as the hub genes identified by both the DEG-PPI network and the WGCNA method. **(B)** The hub genes in DEGs and the yellow module are shown using a Venn diagram. No key genes were identified. **(C)** The hub genes in DEGs and the blue module are shown using a Venn diagram. Seven key genes were identified.

In the present study, because we found there was an ideal overlap in the blue module with the top 30 hub DEGs, the yellow module was significantly positively associated with the DLB group, and we finally chose blue and yellow modules for further study. The scattered plots of blue and yellow modules (Supplementary Figures S4A, B) present significantly positive correlations (*P* < 0.01) between PDD and DLB. We identified 815 and 3 hub genes in the blue and yellow modules, respectively (Supplementary Table S2). Protein interactions among the 1,864 DEGs were predicted using the STRING-DB tool. The top 30 hub genes in DEGs were evaluated by the maximal clique centrality method with the Cytohubba plugin. The network of the top 30 hub genes and expanded DEGs included 234 nodes and 878 edges, visualized by Cytoscape software (Figure 6A). We intersected the hub genes of the blue module and the top 30 hub genes in DEGs (Figures 6B, C), and we identified seven overlapping hub genes. There was no overlap between the top 30 hub genes in DEGs and the hub genes of the yellow module. As shown in Table 1, the information about these seven overlapping hub genes included in the blue module (*SNAP25, 163 GRIN2A, GABRG2, GABRA1, GRIA1, SLC17A6*, and *SYN1*) is mentioned in more detail.

**Table 1:** The information on seven key genes.

**Symbols**	**Full name**	**logFC**	***P*-value**	**Change**	**MM**	**GS**	**Module**
SNAP25	synaptosome associated protein 25	1.37	0.02310	UP	0.916355	0.431507	Blue
GRIN2A	glutamate ionotropic receptor NMDA type subunit 2A	1.46	0.01190	UP	0.938563	0.472282	Blue
GABRG2	gamma-aminobutyric acid type A receptor subunit gamma2	1.63	0.00386	UP	0.937568	0.532672	Blue
GABRA1	gamma-aminobutyric acid type A receptor subunit alpha1	2.12	0.00895	UP	0.922775	0.487116	Blue
GRIA1	glutamate ionotropic receptor AMPA type subunit 1	1.02	0.00722	UP	0.806288	0.505067	Blue
SLC17A6	solute carrier family 17 member 6	1.82	0.00997	UP	0.864285	0.481604	Blue
SYN1	synapsin I	1.09	0.04010	UP	0.876074	0.394251	Blue

Seven key genes were obtained from the intersection of 30 identified genes in 1,864 differentially expressed genes and 815 hub genes in the blue module. The seven key genes were upregulated in the Parkinson's disease dementia group.

## 4. Discussion

The significantly enriched entries for GO and KEGG enrichment analyses demonstrated that the 1,864 DEGs mainly enriched the functions of vesicles and synapses in neurodegenerative diseases. This study supports the mechanistic role of disturbed vesicle trafficking in neurodegenerative diseases (23). Positron emission computed tomography-based study suggests that the loss of synaptic density contributes to dysfunction and cognitive decline in patients with LBD (4). Presynaptic and postsynaptic proteins modulate axonal/dendritic growth and remodeling, thus representing likely key players in the synaptic dysfunction in neurodegenerative diseases (5). Synaptic disruption is a key pathophysiological mechanism leading to neurodegeneration (24). DLB and PDD differ in terms of not only the time of onset of cognitive deficits but also variability in affected functions (2). Patients with DLB present more severe and widespread cognitive dysfunction than those with PDD, particularly in attentive and visuospatial domains, executive functions, constructional tasks (25, 26), and episodic verbal memory (27). Hence, the Mini-Mental State Examination score is lower in patients with DLB than in those with PDD (28). The percentage of patients with DLB who fail to finish the Montreal Cognitive Assessment subitem analysis on the Digit Span Forward was higher than that of patients with PDD, possibly because the former is associated with a more severe attentive deficit than the latter (2). This may explain the enrichment of DEGs.

Due to acting in contrast to each other, DLB and PDD have opposite correlations to enriched pathways as shown in Figure 3. The GSEA data suggested that glycerolipid metabolism and viral myocarditis were positively correlated with PDD. Lipid metabolic dysregulation is involved in the pathogenesis of PDD; α-synuclein may induce dementia in patients with PD possibly through lipid metabolism (29). Coxsackievirus B3 is considered the dominant etiological agent of viral myocarditis. Coxsackievirus B3 infection can induce α-synuclein-associated inclusion body formation in neurons, which might act as a trigger for PD. Transgenic mice that express α-synuclein showed enhanced Coxsackievirus B3 replication and exhibited dopaminergic neuronal death in the substantia nigra (30). A B-cell receptor signaling pathway and one carbon pool by folate signaling pathways were positively correlated with the DLB subset. Accumulating evidence suggests the involvement of immune mediators in DLB (31, 32). Immunization of mice with different B-cell epitopes of human α-synuclein vaccines produced high titers of anti-human α-synuclein antibodies that bound to Lewy bodies and Lewy neurites in the brain tissue of patients with DLB and induced robust helper T-cell expression. Immunotherapeutic approaches that reduce α-synuclein deposits may provide therapeutic benefits for patients with DLB (33). Folate is an essential factor involved in nucleotide synthesis, one-carbon metabolism, and DNA methylation, which have been linked to cognitive impairment and dementia (34). Homocysteine is a central metabolite formed as an intermediate product of one-carbon metabolism, following transmethylation (35). Elevated plasma total homocysteine levels were independently associated with DLB (34).

To further investigate the relationship between co-expressed genes and different LBD subtypes, we performed WGCNA. The results of enrichment for the blue module included axon, postsynapse, presynapse, and neuron projection development. Axonal and synaptic pathology is an important feature of LBD (36). During the early prodromal phase of PD, synaptic alterations happen before cell death, and these alterations are linked to the synaptic accumulation of toxic α-synuclein, specifically in the presynaptic terminals, which affects neurotransmitter release (37). Generalized synaptic degeneration and loss of synaptic density and connectivity may contribute to dysfunction and cognitive decline in patients with neurodegenerative diseases (4, 5, 38). Reduced expression of synaptic proteins could be an index of the degree of synaptic degeneration in the central nervous system (38). Synaptic proteins reliably discriminated PDD and DLB from controls with high sensitivity and specificity (39). The particular synaptic proteins have an important predictive and discriminative molecular fingerprint in neurodegenerative diseases and could be a potential target for early disease intervention (39). The results of the enrichment for the yellow module included positive regulation of macrophage activation and integrin binding. α-synuclein expressed in neurons is released into the extracellular space and taken up by macrophages and microglia; α-synuclein fibrils are considered to be formed from monomers in macrophages and to spread to neurons to induce α-synuclein aggregation in PD model (40). Our data support the difference among axonal and synaptic, inflammation, and neurodegeneration-multiple diseases.

After taking the intersection of the top 30 hub genes of DEGs and the hub genes in blue modules, seven key genes were identified, including *SNAP25, GRIN2A, GABRG2, GABRA1, GRIA1, SLC17A6*, and *SYN1. SNAP25* and *SYN1* as presynaptic proteins are markers of functional synapses (38, 41, 42). *SYN1* is a phosphoprotein that coats the cytoplasmic side of synaptic vesicles and regulates their trafficking within nerve terminals. Inhibition or knockout of *SYN1* can reduce the density of excitatory and inhibitory synapses and impair both glutamatergic and GABAergic synaptic transmission (43). *SNAP25* is a key adhesion molecule for vesicle docking, trafficking, fusion of membranes, and exocytosis, and it has also been implicated in axonal outgrowth and neurite elongation (5). It has been suggested that *SNAP25* could be an effective and accessible biomarker reflecting synaptic integrity and degeneration in the brain (38, 44). In previous studies, *SNAP25* levels were negatively correlated with cognitive functions (38), and *SNAP25* expression was low in patients with PDD and DLB; however, the decrease was more pronounced in the DLB patient group (45), which is consistent with our study so that *SNAP25* is a known gene that is more relevant to LBD (44). The *GRIN2A* gene encodes a member of the glutamate-gated ion channel protein family. *GRIN2A* was found to play important roles not only in synaptic plasticity but also in learning and memory (46), and spatial or discrimination learning impairments have been observed in mice with *GRIN2A* subunit knockout (47, 48). Studies have also shown that the suppression of *GRIN2A* expression impairs the learning of complex motor skills (49). Dendritic branch pruning along with maturation is accompanied by an elevation in *GRIN2A* levels (50). *GRIN2A* deletion was shown to decrease the total dendritic length and dendritic complexity in the dentate gyrus neurons of the hippocampus located in the inner granular zone (51).

*GRIA1* is a subunit in the α-amino-3-hydroxy-5-methylisoxazole-4-propionic acid subtype of ionotropic glutamate receptors, which is a primary receptor that mediates excitatory synaptic transmission at glutamatergic synapses in the central nervous system and plays key roles in synaptic plasticity, neuronal development, and neurological diseases (52). The α-amino-3-hydroxy-5-methylisoxazole-4-propionic acid subtype of ionotropic glutamate receptors mediates most of the fast postsynaptic responses at glutamatergic synapses (53). Synaptic plasticity relies on the normal integration of glutamate receptors at the postsynaptic density (54). The increased translation of *GRIA1* facilitates certain forms of hippocampus-dependent synaptic plasticity and memory (55–57). GO biological process enrichment analysis showed that *SNAP25, GRIN2A, GRIA1*, and *SYN1* were significantly enriched in learning and memory in our study, further suggesting that they participate in the neurobiological basis of pathogenesis by affecting synapse function. KEGG enrichment analysis of DEGs showed that *SNAP25* and *SLC17A6* were enriched in the synaptic vesicle cycle in our study. *SLC17A6* is responsible for the uploading of glutamate into presynaptic vesicles, while *SLC17A6* is utilized by a majority of cortical and subcortical glutamatergic excitatory neurons (58, 59). Kashani et al. observed that *SLC17A6* downregulation was correlated with the degree of cognitive impairment in Brodmann area 9 in patients with Alzheimer's disease (60); the lower the decline in *SLC17A6* expression, the greater the degree of cognitive impairment. Studies suggest that patients with DLB present more severe and widespread cognitive dysfunction than those with PDD (25–27). Consistent with the conclusions of our study, *GRIA1, GRIN2A*, and *SLC17A6* expressions were more upregulated in patients with PDD than in those with DLB, further genetically explaining the cognitive dysfunction is graver in patients with DLB than in those with PDD. The *GABRA1* and *GABRG2* genes encode subunits of the γ-aminobutyric acid (GABA) type A receptor family (61). *GABRA1* was found to be significantly more downregulated in the postmortem frontal cortices of patients with DLB than in those with neuropathological examination normal control (62). Similarly, *GABRG2* expression was found to be significantly low in symptomatic mouse models of tauopathy (63). Both play an important role in the maintenance of normal synaptic function. RNA-sequencing analysis of mutation of *GABRA1* in zebrafish larval brains identified a marked downregulation of genes encoding inhibitory synaptic components as well as proteins involved in axon guidance. Immunocytochemical analysis revealed a marked decrease in the accumulation of GABA synaptic markers; consistently, transgene *GABRG2* mutation resulted in postsynaptic and presynaptic defects (64, 65). *GABRG2* was found to be associated with suicidal behavior and major depressive disorder (66). Studies indicated that low brain levels of GABA may be related to schizophrenia and psychosis (67–69). Fluctuations in core clinical features of DLB are typically delirium-like, occurring as spontaneous alterations in cognition, attention, and arousal (70), which is different from PDD; similarly, in our study, *GABRA1* and *GABRG2* genes were downregulated in DLB.

These findings suggest the potential roles of *SNAP25, GRIN2A, GABRG2, GABRA1, GRIA1, SLC17A6*, and *SYN1* as biomarkers to distinguish PDD from DLB. The function of the seven hub genes participated in the neurobiological basis of pathogenesis by affecting synaptic function and the GABAergic/glutamatergic neurotransmitters. GO enrichment analysis showed that *SNAP25 was* the core gene participating in the neurobiological basis of pathogenesis by affecting synapse function in our study; KEGG enrichment analysis of DEGs showed that *SNAP25* was enriched in the synaptic vesicle cycle. *SNAP25* may be a more significant gene distinguishing between PDD and DLB by the affected synaptic vesicle cycle. In addition, *GRIN2A, GABRG2, GABRA1, GRIA1*, and *SYN1* are involved in regulated GABAergic/glutamatergic functions. Hence, synaptic transmission impairment and GABAergic/glutamatergic dysfunction may be the more outstanding difference between PDD and DLB.

## 5. Supplementary content

We also constructed sets of gene maps based on the online resource OMIM.org (https://omim.org/) (71) associated with DLB and PDD, compared the two lists of gene maps with the top 30 hub genes in DEGs and hub genes in the blue module, respectively, the results suggested that the three lists had no intersection. There were 15 (*GDAP1, ATP1A1, DNAJC6, SNCA, TUBA4A, VCP, GYG1, TRIM2, BMPR2, NEFL, C9orf72, DHX16, FIG4*, and *COPA*) overlap hub genes with the blue module and gene map in PDD (Supplementary Figure S5A; Supplementary Table S3), there were four (*CAMTA1, EXOC6B, ATP6V1B1*, and *SFPQ*) overlap hub genes with the blue module and gene map in DLB (Supplementary Figure S5B; Supplementary Table S4).

## Data availability statement

The original contributions presented in the study are included in the article/Supplementary material, further inquiries can be directed to the corresponding author.

## Ethics statement

Ethical review and approval was not required for the study on human participants in accordance with the local legislation and institutional requirements. Written informed consent from the patients/participants or patients/participants' legal guardian/next of kin was not required to participate in this study in accordance with the national legislation and the institutional requirements.

## Author contributions

J-jC was the senior author of the report. JX made the material preparation and the first draft of the manuscript. All authors contributed to the study's conception, design, read, and approved the final manuscript.

## References

[1] Walker Z Possin KL Boeve BF Aarsland D. Lewy body dementias. Lancet. (2015) 386:1683–97. 10.1016/S0140-6736(15)00462-6 26595642 PMC5792067

[2] Martini A Weis L Schifano R Pistonesi F Fiorenzato E Antonini A. Differences in cognitive profiles between Lewy body and Parkinson's disease dementia. J Neural Transm. (2020) 127:323–30. 10.1007/s00702-019-02129-2 31898759

[3] Jellinger KA. Dementia with Lewy bodies and Parkinson's disease-dementia: current concepts and controversies. J Neural Transm (Vienna). (2018) 125:615–50. 10.1007/s00702-017-1821-9 29222591

[4] Andersen KB Hansen AK Damholdt MF Horsager J Skjaerbaek C Gottrup H. Reduced synaptic density in patients with lewy body dementia: an [11 C]UCB-J PET imaging study. Mov Disord. (2021) 36:2057–65. 10.1002/mds.28617 33899255

[5] Mazzucchi S Palermo G Campese N Galgani A Della Vecchia A Vergallo A. The role of synaptic biomarkers in the spectrum of neurodegenerative diseases. Expert Rev Proteomics. (2020) 17:543–59. 10.1080/14789450.2020.1831388 33028119

[6] Overk CR Masliah E. Pathogenesis of synaptic degeneration in Alzheimer's disease and Lewy body disease. Biochem Pharmacol. (2014) 88:508–16. 10.1016/j.bcp.2014.01.015 24462903 PMC3973539

[7] Zhan SS Beyreuther K Schmitt HP. Quantitative assessment of the synaptophysin immuno-reactivity of the cortical neuropil in various neurodegenerative disorders with dementia. Dementia. (1993) 4:66–74. 10.1159/000107299 8358515

[8] Taylor JP McKeith IG Burn DJ Boeve BF Weintraub D Bamford C. New evidence on the management of Lewy body dementia. Lancet Neurol. (2020) 19:157–69. 10.1016/S1474-4422(19)30153-X 31519472 PMC7017451

[9] Low C Lee JH Lim F Lee C Ballard C Francis PT. Isoform-specific upregulation of FynT kinase expression is associated with tauopathy and glial activation in Alzheimer's disease and Lewy body dementias. Brain Pathol. (2021) 31:253–66. 10.1111/bpa.12917 33128789 PMC8017997

[10] Rajkumar AP Bidkhori G Shoaie S Clarke E Morrin H Hye A. Postmortem cortical transcriptomics of lewy body dementia reveal mitochondrial dysfunction and lack of neuroinflammation. Am J Geriatr Psychiatry. (2019) 28:75–86. 10.1016/j.jagp.06,007. 31327631

[11] Quan W Li J Jin X Liu L Zhang Q Qin Y. Identification of potential core genes in parkinson's disease using bioinformatics analysis. Parkinsons Dis. (2021) 1690341. 10.1155./2021/1690341 34580608 PMC8464436

[12] Chai YL Chong JR Weng J Howlett D Halsey A Lee JH. Lysosomal cathepsin D is upregulated in Alzheimer's disease neocortex and may be a marker for neurofibrillary degeneration. Brain Pathol. (2019) 29:63–74. 10.1111/bpa.12631 30051532 PMC8028263

[13] Xia P Chen J Bai X Li M Wang L Lu Z. Key gene network related to primary ciliary dyskinesia in hippocampus of patients with Alzheimer's disease revealed by weighted gene co-expression network analysis. BMC Neurol. (2022) 22:198. 10.1186/s12883-022-02724-z 35637434 PMC9150314

[14] Zhou Y Zhou B Pache L Chang M Khodabakhshi AH Tanaseichuk O. Metascape provides a biologist-oriented resource for the analysis of systems-level datasets. Nat Commun. (2019) 10:1523. 10.1038/s41467-019-09234-6 30944313 PMC6447622

[15] Yang X Li L Xu C Pi M Wang C Zhang Y. Analysis of the different characteristics between omental preadipocytes and differentiated white adipocytes using bioinformatics methods. Adipocyte. (23945) 11:227–38. 10.1080/2162022, 2063471. 35499169 PMC9067510

[16] Subramanian A Tamayo P Mootha VK Mukherjee S Ebert BL Gillette MA. Gene set enrichment analysis: a knowledge-based approach for interpreting genome-wide expression profiles. Proc Natl Acad Sci. (2005) 102:15545–50. 10.1073/pnas.0506580102 16199517 PMC1239896

[17] Langfelder P Horvath S. WGCNA: an R package for weighted correlation network analysis. BMC Bioinform. (2008) 9:559. 10.1186/1471-2105-9-559 19114008 PMC2631488

[18] Guo Z Zhang Y Ming Z Hao Z Duan P. Identification of key genes in severe burns by using weighted gene coexpression network analysis. Comput Math Methods Med. (2022) 2022:5220403. 10.1155/2022/5220403 35799661 PMC9256319

[19] Zhang R Chen Y He J Gou HY Zhu YL Zhu YM. WGCNA combined with GSVA to explore biomarkers of refractory neocortical epilepsy IBRO. Neurosci Rep. (2022) 13:314–21. 10.1016/j.ibneur.09010 36247523 PMC9561751

[20] Chen G Chen D Feng Y Wu W Gao J Chang C. Identification of key signaling pathways and genes in eosinophilic asthma and neutrophilic asthma by weighted gene co-expression network analysis. Front Mol Biosci. (2022) 9:805570. 10.3389/fmolb.2022.805570 35187081 PMC8847715

[21] Szklarczyk D Gable AL Nastou KC Lyon D Kirsch R Pyysalo S. The STRING database in 2021: customizable protein-protein networks, and functional characterization of user-uploaded gene/measurement sets. Nucleic Acids Res. (2021) 49:D605–12. 10.1093/nar/gkaa1074 33237311 PMC7779004

[22] Yu G Wang LG Han Y He QY. Cluster profiler: an R package for comparing biological themes among gene clusters. OMICS. (2012) 16:284–7. 10.1089/omi.2011.0118 22455463 PMC3339379

[23] Grochowska MM Carreras Mascaro A Boumeester V Natale D Breedveld GJ Geut H. LRP10 interacts with SORL1 in the intracellular vesicle trafficking pathway in non-neuronal brain cells and localises to Lewy bodies in Parkinson's disease and dementia with Lewy bodies. Acta Neuropathol. (2021) 142:117–37. 10.1007/s00401-021-02313-3 33913039 PMC8217053

[24] Jellinger KA Korczyn AD. Are dementia with Lewy bodies and Parkinson's disease dementia the same disease?. BMC Med. (2018) 16:1016. 10.1186./s12916-018-1016-8 29510692 PMC5840831

[25] Petrova M Mehrabian-Spasova S Aarsland D Raycheva M Traykov L. Clinical and neuropsychological differences between mild Parkinson's disease dementia and dementia with lewy bodies. Dement Geriatr Cogn Dis Extra. (2015) 5:212–20. 10.1159/000375363 26195977 PMC4483490

[26] Takemoto M Sato K Hatanaka N Yamashita T Ohta Y Hishikawa N. Different clinical and neuroimaging characteristics in early stage parkinson's disease with dementia and dementia with Lewy bodies. J Alzheimers Dis. (2016) 52:205–11. 10.3233/JAD-150952 27060948 PMC4927815

[27] Park KW Kim HS Cheon SM Cha JK Kim SH Kim JW. Dementia with Lewy bodies versus Alzheimer's disease and parkinson's disease dementia: a comparison of cognitive profiles. J Clin Neurol. (2011) 7:19–24. 10.3988/jcn.71.19 21519522 PMC3079155

[28] Hansen D Ling H Lashley T Foley JA Strand C Eid TM. Novel clinicopathological characteristics differentiate dementia with Lewy bodies from Parkinson's disease dementia. Neuropathol Appl Neurobiol. (2021) 47:143–56. 10.1111/nan.12648 32720329

[29] Dong MX Wei YD Hu L. Lipid metabolic dysregulation is involved in Parkinson's disease dementia. Metab Brain Dis. (2021) 36:463–70. 10.1007/s11011-020-00665-5 33433787

[30] Park SJ Jin U Park SM. Interaction between coxsackievirus B3 infection and α-synuclein in models of Parkinson's disease. PLoS Pathog. (2021) 17:e1010018. 10.1371/journal.ppat.1010018 34695168 PMC8568191

[31] Surendranathan A Rowe JB O'Brien JT. Neuroinflammation in Lewy body dementia Parkinsonism. Relat Disord. (2015) 21:1398–406. 10.1016/j.parkreldis.10009 26493111

[32] Krot M Rolls A. Autoimmunity in neurodegeneration. Science. (2021) 374:823–4. 10.1126/science.abm4739 34762456

[33] Ghochikyan A Petrushina I Davtyan H Hovakimyan A Saing T Davtyan A. Immunogenicity of epitope vaccines targeting different B cell antigenic determinants of human α-synuclein: feasibility study. Neurosci Lett. (2013) 560:86–91. 10.1016/j.neulet.12028 24361548 PMC3928627

[34] Zhang G Liu S Chen Z Shi Z Hu W Ma L. Association of elevated plasma total homocysteine with dementia with Lewy bodies: a case-control study. Front Aging Neurosci. (2021) 13:724990. 10.3389/fnagi.2021.724990 34720990 PMC8555428

[35] Kalecký K Ashcraft P Bottiglieri T. One-carbon metabolism in Alzheimer's disease and Parkinson's disease brain tissue. Nutrients. (2022) 14:599. 10.3390/nu14030599 35276958 PMC8838558

[36] Xing H Lim YA Chong JR Lee JH Aarsland D Ballard CG. Increased phosphorylation of collapsin response mediator protein-2 at Thr514 correlates with β-amyloid burden and synaptic deficits in Lewy body dementias. Mol Brain. (2016) 9:84. 10.1186/s13041-016-0264-9 27609071 PMC5016931

[37] Cardinale A Calabrese V Iure de Picconi A. Alpha-synuclein as a prominent actor in the inflammatory synaptopathy of Parkinson's disease. Int J Mol Sci. (2021) 22:6517. 10.3390/ijms22126517 34204581 PMC8234932

[38] Agliardi C Guerini FR Zanzottera M Bianchi A Nemni R Clerici M. SNAP-25 in serum is carried by exosomes of neuronal origin and is a potential biomarker of Alzheimer's disease. Mol Neurobiol. (2019) 56:5792–8. 10.1007/s12035-019-1501-x 30680692

[39] Bereczki E Branca RM Francis PT Pereira JB Baek JH Hortobágyi T. Synaptic markers of cognitive decline in neurodegenerative diseases: a proteomic approach. Brain. (2018) 141:582–95. 10.1093/brain/awx352 29324989 PMC5837272

[40] Moriya S Hanazono M Fukuhara T Iwase K Hattori N Takiguchi M. A53T. mutant α-synuclein fibrils formed in macrophage are spread to neurons. Cell Mol Life Sci. (2022) 79:234. 10.1007/s00018-022-04263-9 35397671 PMC11073293

[41] VanGuilder HD Farley JA Yan H Van Kirk CA Mitschelen M Sonntag WE. Hippocampal dysregulation of synaptic plasticity-associated proteins with age-related cognitive decline. Neurobiol Dis. (2011) 43:201–12. 10.1016/j.nbd.03012 21440628 PMC3096728

[42] Taniguchi K Yamamoto F Amano A Tamaoka A Sanjo N Yokota T. Amyloid-β oligomers interact with NMDA receptors containing GluN2B subunits and metabotropic glutamate receptor 1 in primary cortical neurons: Relevance to the synapse pathology of Alzheimer's disease. Neurosci Res. (2022) 180:90–8. 10.1016/j.neures.03001 35257837

[43] Rocchi A Sacchetti S De Fusco A Giovedi S Parisi B Cesca F. Autoantibodies to synapsin I sequestrate synapsin I and alter synaptic function. Cell Death Dis. (2019) 10:64. 10.1038/s41419-019-2106-z 31727880 PMC6856194

[44] Agliardi C Meloni M Guerini FR Zanzottera M Bolognesi E Baglio F. Oligomeric α-Syn and SNARE complex proteins in peripheral extracellular vesicles of neural origin are biomarkers for Parkinson's disease. Neurobiol Dis. (2020) 148:105185. 10.1016/j.nbd.2020.105185 33217562

[45] Bereczki E Francis PT Howlett D Pereira JB Höglund K Bogstedt A. (2016). Synaptic proteins predict cognitive decline in Alzheimer's disease and Lewy body dementia Alzheimers Dement. (2016) 12:1149–58. 10.1016/j.jalz.04,005. 27224930

[46] Sun Y Cheng X Zhang L Hu J Chen Y Zhan L. The functional and molecular properties, physiological functions, and pathophysiological roles of GluN2A in the central nervous system. Mol Neurobiol. (2017) 54:1008–21. 10.1007/s12035-016-9715-7 26797520

[47] Sakimura K Kutsuwada T Ito I Manabe T Takayama C Kushiya E. Reduced hippocampal LTP and spatial learning in mice lacking NMDA receptor epsilon 1 subunit. Nature. (1995) 373:151–5. 10.1038/373151a0 7816096

[48] Brigman JL Feyder M Saksida LM Bussey TJ Mishina M Holmes A. Impaired discrimination learning in mice lacking the NMDA receptor NR2A subunit. Learn Mem. (2008) 15:50–4. 10.1101/lm.777308 18230672 PMC3575092

[49] Lemay-Clermont J Robitaille C Auberson YP Bureau G Cyr M. Blockade of NMDA receptors 2A subunit in the dorsal striatum impairs the learning of a complex motor skill. Behav Neurosci. (2011) 125:714–23. 10.1037/a0025213 21859173

[50] Bustos FJ Varela-Nallar L Campos M Henriquez B Phillips M Opazo C. PSD95 suppresses dendritic arbor development in mature hippocampal neurons by occluding the clustering of NR2B-NMDA receptors. PLoS ONE. (2014) 9:e94037. 10.1371/journal.pone.0094037 24705401 PMC3976375

[51] Kannangara TS Bostrom CA Ratzlaff A Thompson L Cater RM Gil-Mohapel J. Deletion of the NMDA receptor GluN2A subunit significantly decreases dendritic growth in maturing dentate granule neurons. PLoS ONE. (2014) 9:e103155. 10.1371/journal.pone.0103155 25083703 PMC4118862

[52] Ge Y Wang YT. GluA1-homomeric AMPA receptor in synaptic plasticity and neurological diseases. Neuropharmacology. (2021) 197:108708. 10.1016/j.neuropharm.2021.108708 34274350

[53] Wang GJ Kang L Kim JE Maro GS Xu XZ Shen K. GRLD-1 regulates cell-wide abundance of glutamate receptor through post-transcriptional regulation. Nat Neurosci. (2010) 13:1489–95. 10.1038/nn.2667 21037582 PMC3087617

[54] Gong Y Lippa CF. Review: disruption of the postsynaptic density in Alzheimer's disease and other neurodegenerative dementias. Am J Alzheimers Dis Other Demen. (2010) 25:547–55. 10.1177/1533317510382893 20858652 PMC2976708

[55] Pavlopoulos E Trifilieff P Chevaleyre V Fioriti L Zairis S Pagano A. Neuralized1 activates CPEB3: a function for nonproteolytic ubiquitin in synaptic plasticity and memory storage. Cell. (2011) 147:1369–83. 10.1016/j.cell.09056 22153079 PMC3442370

[56] Quadri Z Johnson N Zamudio F Miller A Peters M Smeltzer S. Overexpression of human wtTDP-43 causes impairment in hippocampal plasticity and behavioral deficits in CAMKII-tTa transgenic mouse model. Mol Cell Neurosci. (2020) 102:103418. 10.1016/j.mcn.2019.103418 31705957 PMC7505208

[57] Yeung JH Calvo-Flores Guzmán B Palpagama TH Ethiraj J Zhai Y Tate WP. Amyloid-beta1–42 induced glutamatergic receptor and transporter expression changes in the mouse hippocampus. J Neurochem. (2020) 155:62–80. 10.1111/jnc.15099 32491248

[58] Kashani A Betancur C Giros B Hirsch E El Mestikawy S. Altered expression of vesicular glutamate transporters VGLUT1 and VGLUT2 in Parkinson disease. Neurobiol Aging. (2006) 28:568–78. 10.1016/j.neurobiolaging.02010 16563567 PMC1976623

[59] Poirel O Mella S Videau C Ramet L Davoli MA Herzog E. Moderate decline in select synaptic markers in the prefrontal cortex (BA9) of patients with Alzheimer's disease at various cognitive stages. Sci Rep. (2018) 8:938. 10.1038/s41598-018-19154-y 29343737 PMC5772053

[60] Kashani A Lepicard E Poirel O Videau C David JP Fallet-Bianco C. Loss of VGLUT1 and VGLUT2 in the prefrontal cortex is correlated with cognitive decline in Alzheimer disease. Neurobiol Aging. (2007) 29:1619–30. 10.1016/j.neurobiolaging.04010 17531353

[61] Steudle F Rehman S Bampali K Simeone X Rona Z Hauser E. A novel de novo variant of GABRA1 causes increased sensitivity for GABA *in vitro*. Sci Rep. (2020) 10:2379. 10.1038/s41598-020-59323-6 32047208 PMC7012862

[62] Santpere G Garcia-Esparcia P Andres-Benito P Lorente-Galdos B Navarro A Ferrer I. Transcriptional network analysis in frontal cortex in Lewy body diseases with focus on dementia with Lewy bodies. Brain Pathol. (2018) 28:315–33. 10.1111/bpa.12511 28321951 PMC8028609

[63] Jiang S Wen N Li Z Dube U Del Aguila J Budde J. Integrative system biology analyses of CRISPR-edited iPSC-derived neurons and human brains reveal deficiencies of presynaptic signaling in FTLD and PSP. Transl Psychiatry. (2018) 8:265. 10.1038/s41398-018-0319-z 30546007 PMC6293323

[64] Samarut É Swaminathan A Riché R Liao M Hassan-Abdi R Renault S. γ-aminobutyric acid receptor alpha 1 subunit loss of function causes genetic generalized epilepsy by impairing inhibitory network neurodevelopment. Epilepsia. (2018) 59:2061–74. 10.1111/epi.14576 30324621

[65] Zhou J Liang W Wang J Chen J Liu D Wang X. An epileptic encephalopathy associated GABRG2 missense mutation leads to pre- and postsynaptic defects in zebrafish. Hum Mol Genet. (2021) 3:ddab338. 10.1093./hmg/ddab338 34957497

[66] Yin H Pantazatos SP Galfalvy H Huang YY Rosoklija GB Dwork AJ. A pilot integrative genomics study of GABA and glutamate neurotransmitter systems in suicide, suicidal behavior, and major depressive disorder. Am J Med Genet B Neuropsychiatr Genet. (2016) 171:414–26. 10.1002/ajmg.b.32423 26892569 PMC4851346

[67] Blum BP Mann JJ. The GABAergic system in schizophrenia. Int J Neuropsychopharmacol. (2002) 5:159–79. 10.1017/S1461145702002894 12135541

[68] Hoftman GD Volk DW Bazmi HH Li S Sampson AR Lewis DA. Altered cortical expression of GABA-related genes in schizophrenia: illness progression vs developmental disturbance. Schizophr Bulletin. (2015) 41:180–91. 10.1093/schbul/sbt178 24361861 PMC4266281

[69] Quiñones GM Mayeli A Yushmanov VE Hetherington HP Ferrarelli F. Reduced GABA/glutamate in the thalamus of individuals at clinical high risk for psychosis. Neuropsychopharmacology. (2021) 46:1133–9. 10.1038/s41386-020-00920-4 33273706 PMC8115482

[70] McKeith IG Boeve BF Dickson DW Halliday G Taylor JP Weintraub D. Diagnosis and management of dementia with Lewy bodies: Fourth consensus report of the DLB Consortium. Neurology. (2017) 89:88–100. 10.1212/WNL.0000000000004058 28592453 PMC5496518

[71] Amberger JS Bocchini CA Scott AF Hamosh A. OMIM.org: leveraging knowledge across phenotype-gene relationships. Nucleic Acids Res. (2019) 47:D1038–43. 10.1093/nar/gky1151 30445645 PMC6323937

