# Supplementary figures and images for: Identification of key genes and signaling pathways associated with dementia with Lewy bodies and Parkinson's disease dementia using bioinformatics
Source: Front Neurol. 2023 Mar 9;14:1029370. doi: 10.3389/fneur.2023.1029370 (PMC10034123; doi:10.3389/fneur.2023.1029370)

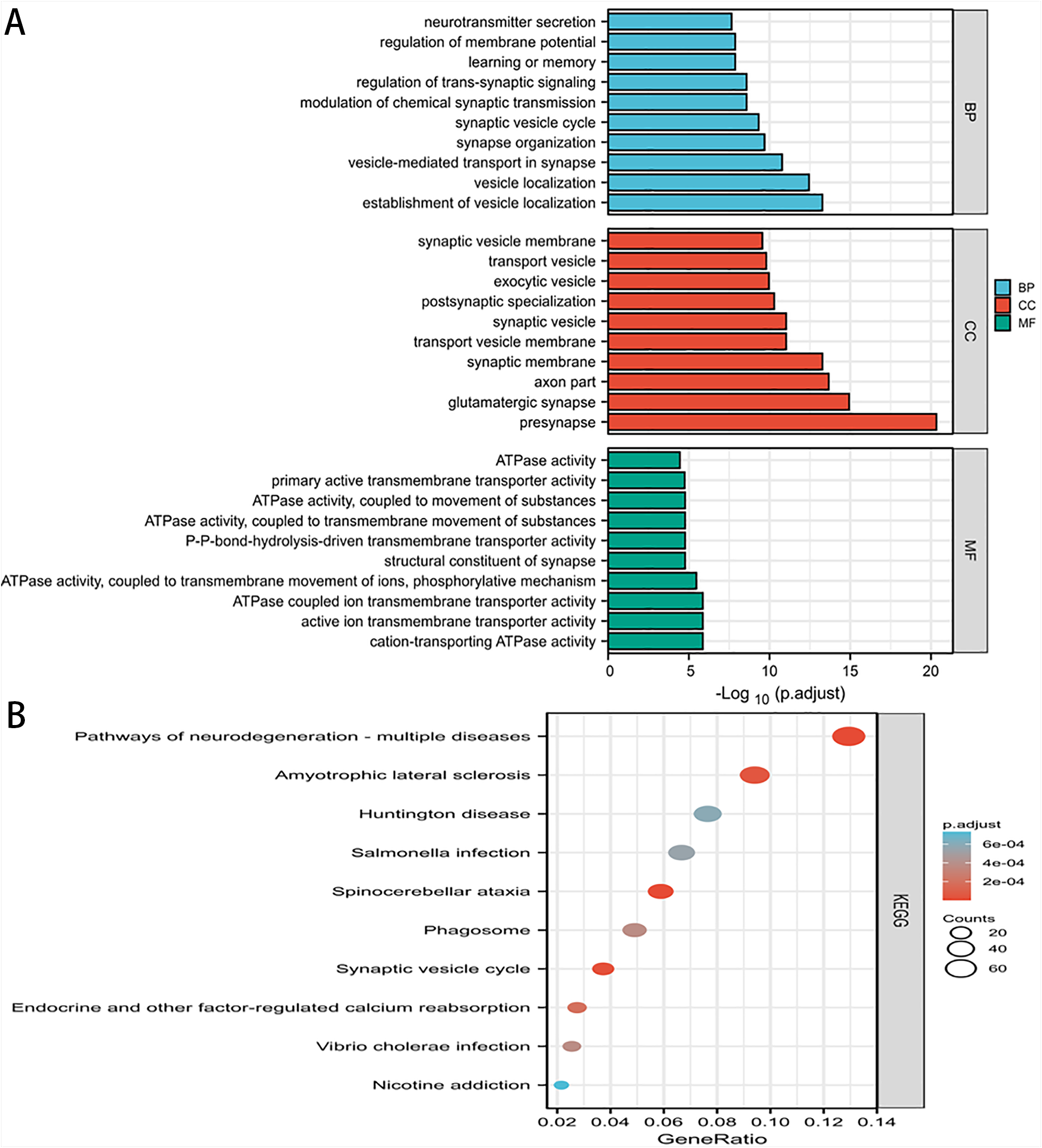

Supplement: Supplementary file 1 [file Data_Sheet_1.ZIP › Figure S1.tif]

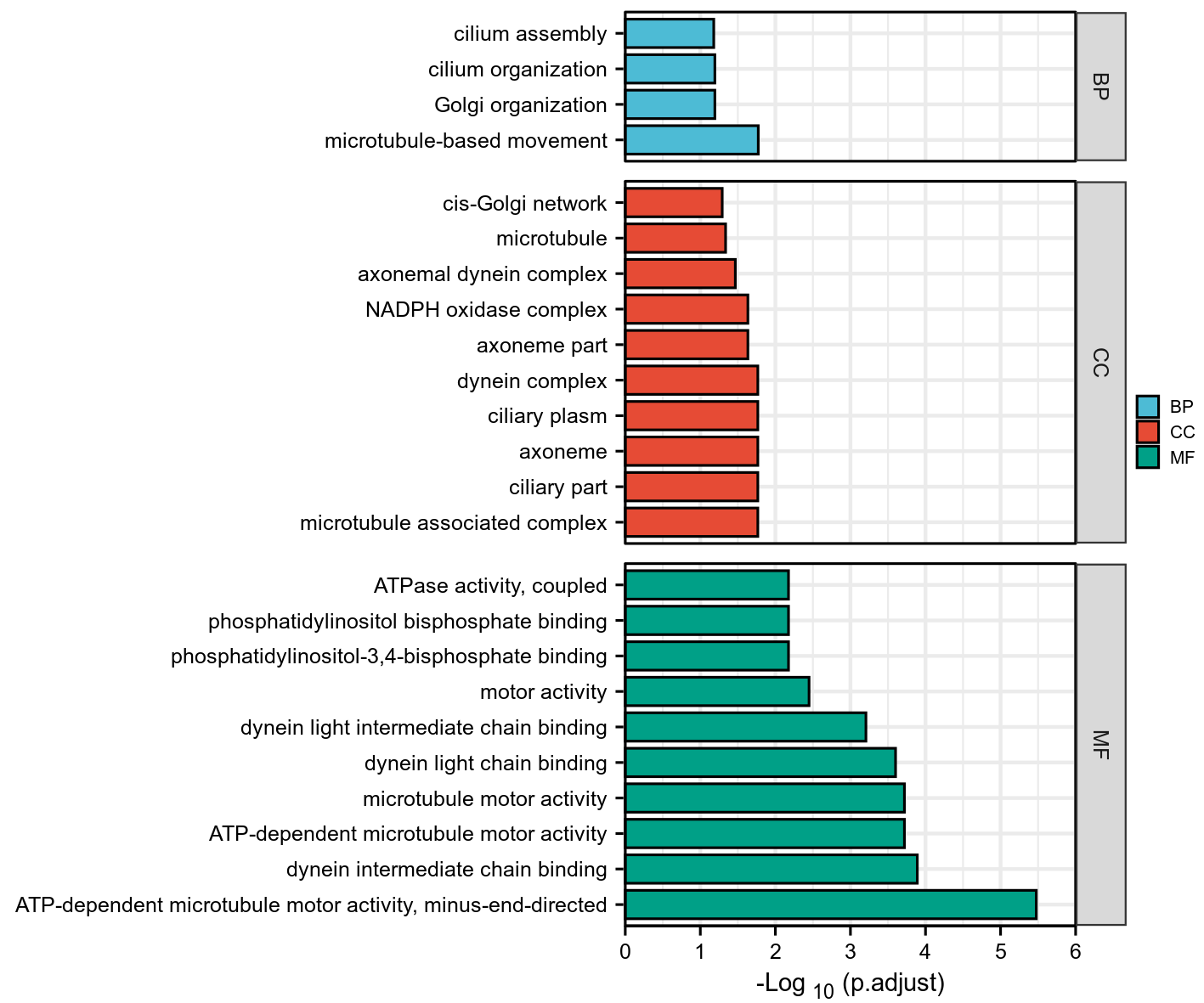

Supplement: Supplementary file 1 [file Data_Sheet_1.ZIP › Figure S2.tiff]

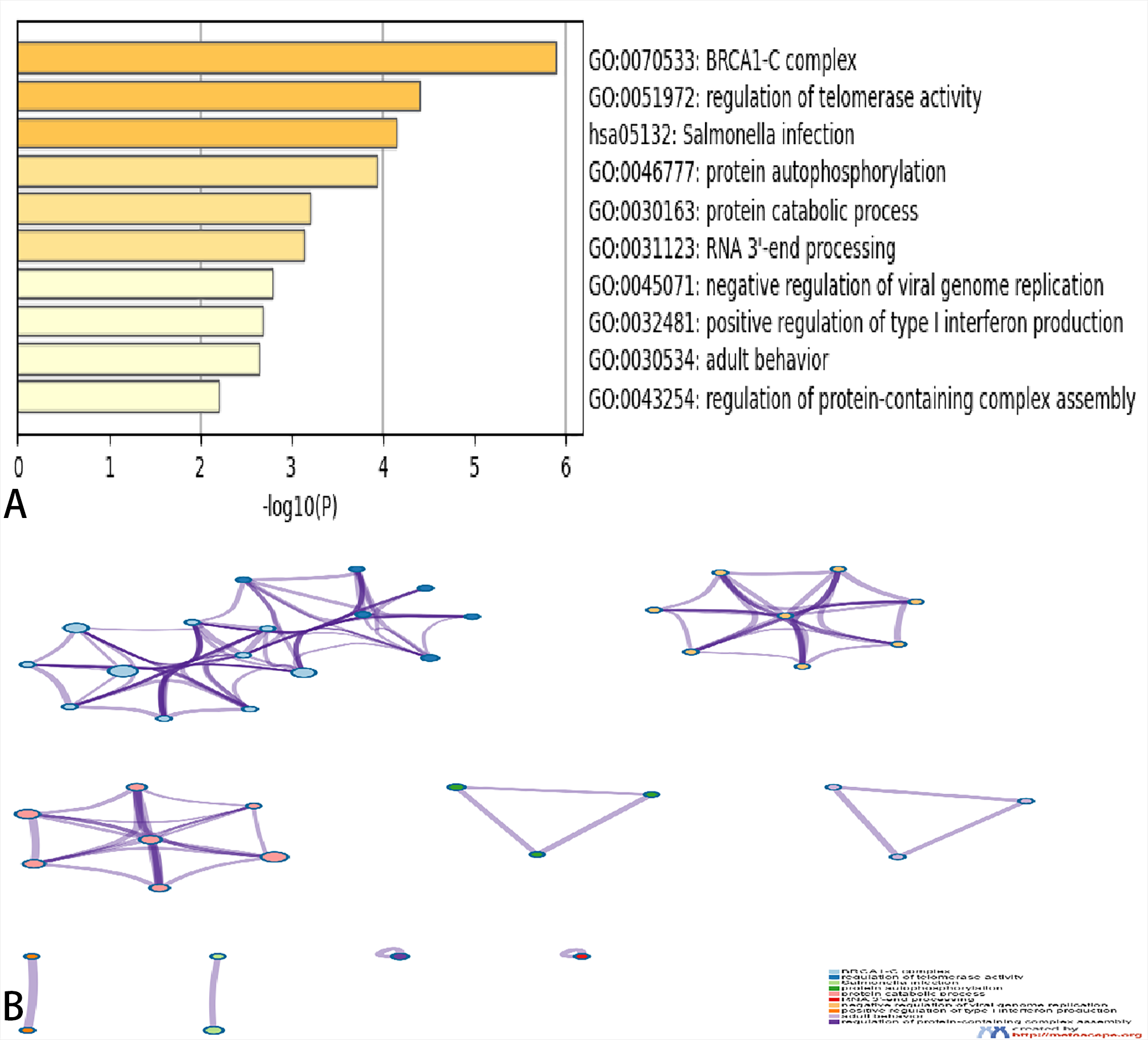

Supplement: Supplementary file 1 [file Data_Sheet_1.ZIP › Figure S3.tif]

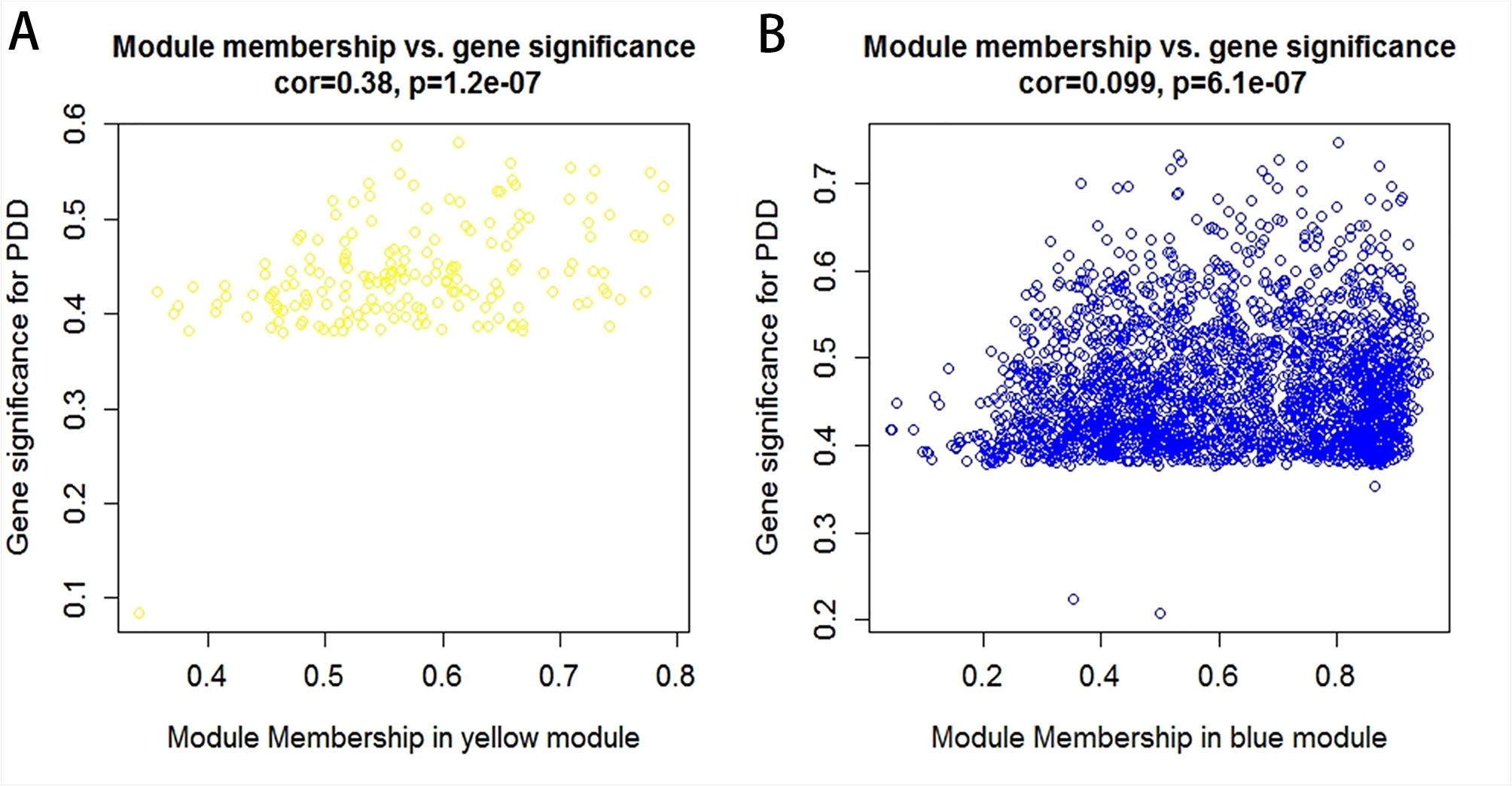

Supplement: Supplementary file 1 [file Data_Sheet_1.ZIP › Figure S4.tif]

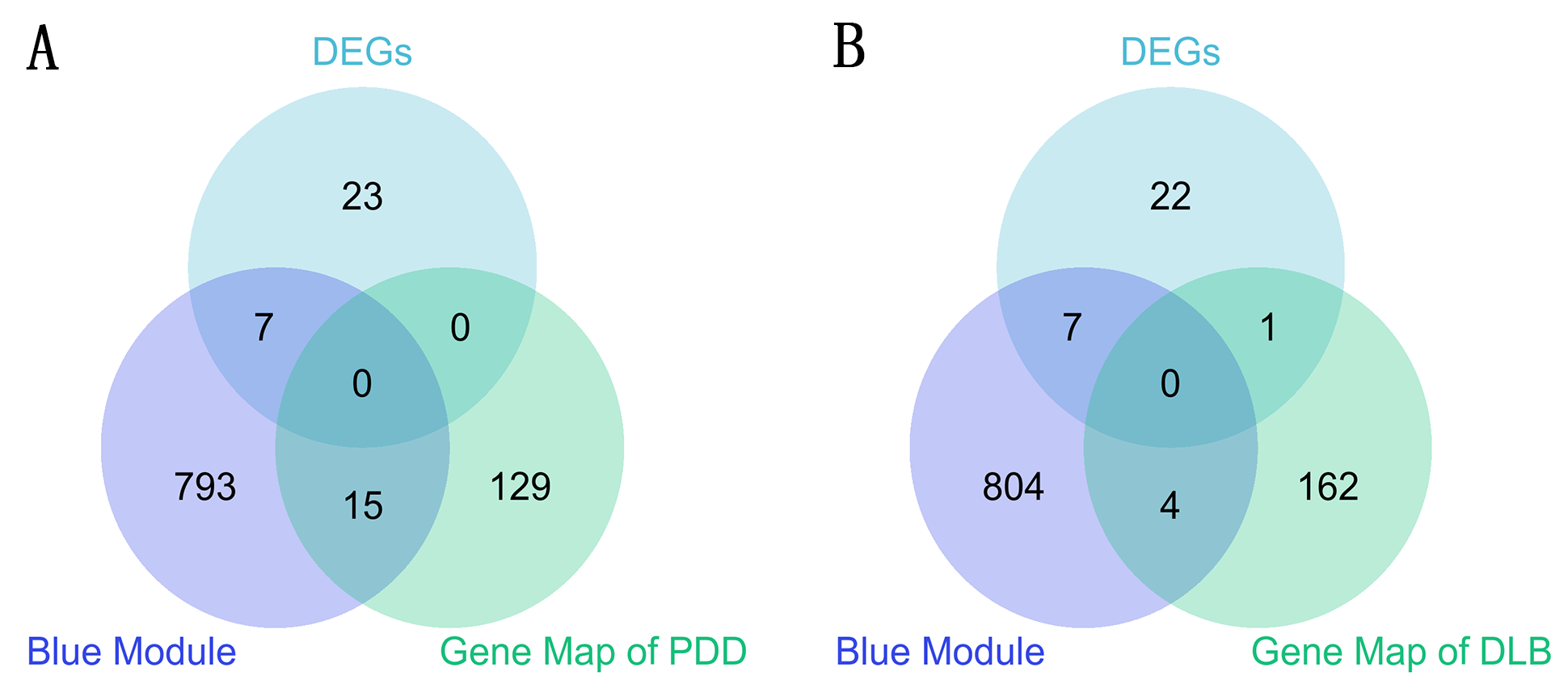

Supplement: Supplementary file 1 [file Data_Sheet_1.ZIP › Figure S5.tif]
